# Bidirectional longitudinal associations between loneliness and pain, and the role of inflammation

**DOI:** 10.1097/j.pain.0000000000002082

**Published:** 2020-09-18

**Authors:** Anna Loeffler, Andrew Steptoe

**Affiliations:** a Department of Behavioural Science and Health, University College London, London, United Kingdom; b Institute for Social Science Research, University of Queensland, Brisbane, Queensland, Australia

**Keywords:** Loneliness, Chronic pain, Longitudinal, C-Reactive protein

## Abstract

Pain and loneliness are consistently associated, but the direction of the relationship is uncertain. We assessed bidirectional associations over a 4-year period in a sample of 4906 men and women (mean 65.1 ± 8.72 years) who were participants in the English Longitudinal Study of Ageing. The role of inflammation in these links was also investigated. Pain was defined by reports of being often troubled by pain at a moderate or severe intensity, whereas loneliness was measured using the shortened UCLA scale. Age, sex, ethnicity, educational attainment, wealth as a marker of socioeconomic resources, marital status, physical activity, and depressive symptoms were included as covariates. We found that baseline loneliness was associated with pain 4 years later after adjusting for baseline pain and other covariates (odds ratio [OR] = 1.25, 95% confidence interval [CI] 1.06-1.47, *P* = 0.007). Similarly, baseline pain independently predicted loneliness 4 years later (OR = 1.34, 95% CI 1.14-1.58, *P* = 0.001). Associations remained significant after additional adjustment for baseline mobility impairment. Likelihood of pain on follow-up was heightened when baseline loneliness was accompanied by elevated C-reactive protein concentration (OR = 1.50, 95% CI 1.13-2.00, *P* = 0.006), whereas inflammation did not predict future loneliness or contribute to the association between baseline pain and future loneliness. Both pain and loneliness are distressing experiences that impact well-being and quality of life. We conclude that there were bidirectional longitudinal relationships between pain and loneliness in this representative sample of older men and women, but that the mechanisms underlying these processes may differ.

## Abstract 2

Supplemental Digital Content is Available in the Text.

Bidirectional associations between loneliness and pain over a 4-year period were observed in a sample of 4906 older men and women.

## 1. Introduction

Chronic pain and loneliness are common problems among older adults. Population studies indicate that the prevalence of chronic pain is more than 50% in people aged 65 years and older,^1,14,30^ whereas loneliness contributes to increased risk of physical and cognitive functional decline, mental ill-health, and cardiovascular disease.^3,32,39^ Loneliness and pain are positively associated,^34^ and it has been argued that there are common brain mechanisms underlying physical and social pain.^10,11,15^ However, the temporal relationship between loneliness and pain is uncertain. Pain may limit activity and social engagement, thereby contributing to loneliness, whereas the stress of loneliness may exacerbate pain.

Longitudinal studies are valuable in identifying the temporal sequence. A study of cancer survivors, benign controls, and older adults showed that loneliness predicted increases in pain up to 4 years later independently of sleep problems, physical exercise, and other covariates.^19^ By contrast, an analysis of the Jerusalem Longitudinal Cohort Study found no association between loneliness and chronic pain 7 years later in people aged 70 to 90 years at baseline.^38^ Results for the reverse relationship have been inconsistent as well. An investigation of the Health and Retirement Study over a 4-year period indicated that incident loneliness was predicted by pain, but only if pain was present both at baseline and follow-up.^12^ Another community study documented cross-sectional associations between pain and loneliness, but found that baseline pain was minimally related to future loneliness.^4^

The potentially bidirectional links between pain and loneliness have typically been investigated in separate studies, making it difficult to determine the relative importance of the 2 temporal sequences. We therefore tested associations between loneliness and future pain, and pain and future loneliness, in a single study of a nationally representative population sample involving 4906 men and women aged 52 years and older. We also studied the contribution of a number of factors that potentially explain part of the association. These include socioeconomic status, which is linked both to pain and loneliness, and physical inactivity.^5,29,35^ Another potential confounder is depression because depressive symptoms are closely linked both with loneliness and pain.^7,30^

The second aim of this study was to evaluate the possible role of inflammation in linking pain and loneliness. Inflammation contributes to the experience of many types of disease-related pain, and there are associations between inflammatory biomarkers and nonspecific pain.^23^ Low-grade systemic inflammation may induce sensitization of pain pathways and peripheral nociceptors.^2,16^ There is also evidence that inflammation is related to loneliness, possibly as part of the stress response associated with perceptions of social isolation. Thus, loneliness is associated with proinflammatory gene expression,^8^ and with elevated levels of markers of inflammation such as C-reactive protein (CRP) and interleukin-6.^28,33^ It is therefore conceivable that inflammation constitutes a biological pathway through which loneliness augments risk of future pain, and vice versa. We therefore tested whether heightened inflammation indexed by elevated CRP augmented the association between loneliness and future pain, or the link between pain and future loneliness, 4 years later.

## 2. Methods

Data were analysed from the English Longitudinal Study of Ageing (ELSA) a nationally representative population study of men and women aged 50 years and older living in England.^37^ The study started in 2002, and data are collected every 2 years using face-to-face computer-assisted personal interviews held in participants' homes, coupled with a self-completion questionnaire. The baseline for these analyses was wave 2 (2004) because this was the first wave in which loneliness was assessed. A total of 8039 participants completed measures of loneliness and pain in Wave 2, of whom 5183 also completed assessments 4 years later in Wave 4 (2008). Data on covariates were missing for 277 individuals, therefore the analytic sample was 4906 (2158 men and 2748 women). There were no significant differences between individuals included and excluded from the longitudinal analyses in baseline levels of loneliness or pain. The analyses of the role of inflammation were conducted on 3701 participants who had blood samples obtained during a separate home visit by study nurses. The study was approved through the National Research Ethics Service, and all participants provided informed consent.

### 2.1. Measures

We measured pain by participant self-report, using the questions, “Are you often troubled with pain?” and, if so, “How bad is the pain most of the time?” (with options of mild, moderate, or severe). In line with previous research, we specifically focused on pain that was classed as moderate or severe.^13,42^ Loneliness was measured with the three-item short form of the Revised UCLA loneliness scale.^18^ Each item was scored on a 3-point scale: *hardly ever or never*, *some of the time*, and *often*. We defined those individuals who reported they were lonely some of the time on at least 2 items as lonely. Both pain and loneliness were assessed at baseline (2004) and follow-up (2008).

The covariates in these analyses included sex, age, ethnicity, education, wealth, marital status, physical activity, and depressive symptoms because these factors are potentially associated with chronic pain and loneliness. Age was modelled as a continuous variable. Participants were categorised into white European and other because the number of participants of non-European origin was very small.^37^ Education was classified according to the person's highest educational qualification into 3 categories: low (no qualifications), intermediate (qualifications at the end of state-regulated schooling), and higher (high school graduation up to university degree). Wealth is a robust indicator of economic resources among older people,^9^ and was measured with a detailed assessment of financial, housing and physical wealth (such as land, business wealth, and jewellery), excluding pension wealth. Respondents were divided into married/cohabiting and not married at baseline. Physical activity was assessed by a series of questions concerning frequency of vigorous, moderate, and light activities categorised according to their metabolic equivalent (MET). The vigorous activities corresponded to MET ≥ 6, moderate to ≥3.5 and <6, and light ≥ 2 to <3.5. They were subsequently classified into 5 levels: level 0—no moderate and no vigorous activity; level 1—Moderate activity once a week or 1 to 3 times a month and no vigorous activity; level 2—moderate activity more than once a week and no vigorous activity, or vigorous activity 1 to 3 times a month and no moderate activity; level 3—moderate activity once a week or more and vigorous activity once a week or 1 to 3 times a month, or vigorous activity once a week and moderate activity 1 to 3 times a month or never, or moderate activity 1 to 3 times a month and vigorous activity 1 to 3 times a month; level 4—vigorous activity more than once a week, with or without moderate activity.^26^ Depressive symptoms were measured using the 8-item Center for Epidemiologic Studies Depression Scale (CES-D),^36^ a shortened scale with a Cronbach α of 0.78 in this sample. The item on loneliness was excluded,^6^ and we used a score of ≥3 to indicate the presence of significant depressive symptoms.^31^

High-sensitivity plasma CRP concentration was assayed from blood samples obtained during study nurse visits to participants' homes at baseline. We classified individuals with values ≥ 3 mg/L as having raised CRP levels because this is an established threshold in population and clinical studies. Results were the same when CRP was analysed as a continuous variable. Individuals with values ≥ 20 mg/L were excluded because high values may indicate the presence of an acute infection or serious acute illness.

In sensitivity analyses, we also included mobility impairment as a covariate. Participants were asked about their ability to perform 10 tasks such as climbing a flight of stairs without resting, or picking up a small coin. They were subsequently classified into those who did or did not experience any mobility impairment at baseline.

### 2.2. Statistical analysis

Data were analysed using a series of multivariable logistic regressions, and results are presented as odds ratios (ORs) with 95% confidence intervals (CI). In longitudinal analysis, we first assessed whether loneliness at baseline was a predictor of chronic pain 4 years later, independently of baseline chronic pain and other covariates. We therefore computed the adjusted odds of reporting chronic pain on follow-up with the low loneliness group as the reference category. Four models were tested. Model 1 included age, sex along with loneliness as determinants of future pain. In model 2, we added baseline pain to the regression. Ethnicity, education, wealth, marital status, and physical activity were added in model 3 to discover the extent to which these factors explained associations between loneliness and future pain. Depressive symptoms were added in model 4 to detect the extent to which the association of loneliness with future pain was independent of depression.

A second set of analyses restricted analysis to people who had no pain at baseline, so tested the association between baseline loneliness and incident pain using the same set of models as described above. We also computed the proportion of people with and without loneliness at baseline who developed chronic pain over the follow-up period.

A parallel strategy was used to analyse the association between chronic pain and future loneliness. Model 1 included age, sex, and baseline chronic pain as determinants of loneliness 4 years later. Model 2 added baseline loneliness, whereas ethnicity, education, wealth, marital status, and physical activity were included in model 3. Depressive symptoms were added in model 3. The analysis of incident loneliness tested the association between baseline pain and the development of loneliness among people who were not lonely at baseline.

In sensitivity analyses, we repeated both the regressions on future chronic pain and future loneliness, adding mobility impairment as an additional covariate to the fully adjusted models. Interactions with inflammation were tested by categorising respondents into groups of low or high baseline loneliness and low or high CRP for the analyses of chronic pain at 4 years. The adjusted ORs of pain for each category were assessed with the low lonely/low CRP group as the reference category. Likewise, we classified people into baseline pain or no pain and low or high CRP for the analyses of loneliness at 4 years, estimating the adjusted OR of loneliness with the no pain/low CRP group as the reference category.

## 3. Results

The study sample comprised 2158 men and 2748 women with an average age of 65.1 ± 8.72 years. The large majority were of white European background with moderate levels of education; less than one-third had college education (Table 1). Men tended to be more educated, wealthier, and more likely to be married than women. Significant depressive symptoms were present in 11.3% of participants overall, and were more common among women. Physical activity levels were significantly higher in men than women. Around 30% reported moderate or high loneliness, whereas 24.6% experienced moderate/severe pain at baseline. Over the 4-year follow-up period, 16.5% of respondents with no pain at baseline reported moderate/severe pain, whereas 37.1% who were in pain at baseline were no longer experiencing pain on follow-up. The incidence of loneliness among people who were not lonely at baseline was 17.4%, whereas 33.5% of lonely people at baseline were no longer lonely 4 years later. There were significant sex differences in loneliness and pain, with women reporting higher levels than men. Overall, 31.9% of participants had CRP concentrations ≥ 3.0 mg/L, with higher rates among women. Cross-sectionally, loneliness at baseline was positively associated with moderate/severe pain after adjustment for age, sex, ethnicity, education, wealth, marital status, physical activity, and depression (partial *r* = 0.063, *P* <0.001).

**Table 1:** Participant characteristics.

	Total (n = 4905)	Men (n = 2156)	Women (n = 2749)	*P* Difference
Age (y)	65.11 ± 8.72	64.86 ± 8.45	65.31 ± 8.92	0.074
Ethnicity (white European)	4853 (98.9%)	2133 (98.8%)	2720 (99.0%)	0.68
Education				<0.001
Lower	1586 (32.3%)	559 (25.9%)	1027 (37.4%)	
Intermediate	1950 (39.7%)	815 (37.8%)	1135 (41.3%)	
Higher	1370 (27.9%)	784 (36.3%)	586 (21.3%)	
Wealth:				<0.001
Lowest	663 (13.5%)	245 (11.4%)	418 (15.2%)	
2	862 (17.6%)	358 (16.6%)	506 (18.4%)	
3	997 (20.3%)	426 (19.7%)	571 (20.8%)	
4	1142 (23.3%)	531 (24.6%)	611 (22.2%)	
Highest	1240 (25.3%)	598 (27.7%)	642 (23.4%)	
Marital status (married)	3421 (69.7%)	1725 (79.9%)	1696 (61.7%)	<0.001
Depression (significant symptoms)	552 (11.3%)	169 (7.8%)	383 (13.9%)	<0.001
Physical activity	2.28 ± 1.24	2.46 ± 1.20	2.13 ± 1.25	<0.001
Loneliness (wave 2)	1448 (29.5%)	544 (25.3%)	903 (32.9%)	<0.001
Loneliness (wave 4)	1563 (31.9%)	574 (26.6%)	989 (36.0%)	<0.001
Mod/severe pain (wave 2)	1208 (24.6%)	413 (19.1%)	795 (28.9%)	<0.001
Mod/severe pain (wave 4)	1370 (27.9%)	470 (21.8%)	900 (32.8%)	<0.001
C-reactive protein ≥ 3 mg/L	1162 (31.9%)	437 (26.9%)	725 (35.9%)	<0.001

### 3.1. Loneliness as a predictor of future chronic pain

The longitudinal associations between loneliness and pain 4 years later are summarized in Table 2. Loneliness at baseline was related to the presence of moderate/severe pain 4 years later independently of age and sex (model 1), with an adjusted OR of 1.45 (95% CI 1.25-1.69) when baseline pain had also been taken into account (model 2). The association was reduced to 1.35 after ethnicity, education, wealth, marital status, and physical activity had been taken into account (model 3), and fell further after the inclusion of depressive symptoms in the regression (model 4). Nonetheless, the association remained significant, with 25% higher adjusted odds of moderate/severe pain per unit increase in loneliness. The full details of regression model 4 are provided in Supplementary Table 1 (available at http://links.lww.com/PAIN/B182). Other independent determinants of pain after 4 years were baseline pain, female sex, lesser wealth, physical inactivity, being married, and depressive symptoms at baseline. In sensitivity analysis including baseline mobility impairment as a covariate, the association between loneliness and follow-up pain remained significant (adjusted OR = 1.19, 95% CI 1.01-1.40, *P* = 0.039).

**Table 2:** Bidirectional associations between loneliness and chronic pain.

	Loneliness as a predictor of future chronic pain		Chronic pain as a predictor of loneliness	
	Adjusted OR (95% CI)	*P*	Adjusted OR (95% CI)	*P*
Model 1				
Adjusted for age and sex	1.74 (1.53-1.99)	<0.001	1.86 (1.62-2.13)	<0.001
Model 2				
Additionally adjusted for baseline pain* or loneliness†	1.45 (1.25-1.69)*	<0.001	1.55 (1.32-1.81)†	<0.001
Model 3				
Additionally adjusted for ethnicity, education, wealth, marital status, and physical activity	1.35 (1.16-1.58)	<0.001	1.42 (1.21-1.67)	<0.001
Model 4				
Additionally adjusted for baseline depressive symptoms	1.25 (1.06-1.47)	0.007	1.34 (1.14-1.58)	0.001

* analyses of future chronic pain.

† analyses of future loneliness.

Adjusted odds ratios (OR) with 95% confidence intervals (CI).

The analysis of incident pain among the 3697 individuals who were free of pain at baseline is summarised in Supplementary Table 2 (available at http://links.lww.com/PAIN/B182). The results are comparable to those in the primary analysis, with an adjusted odds of new pain reports of 1.39 (95% CI 1.13-1.79, *P* = 0.001) for lonely individuals in the fully adjusted model. In absolute terms, this translated into incident pain in 20.0% of participants who were lonely at baseline, compared with 15.2% in those who were not lonely.

### 3.2. Moderate/severe pain as a predictor of future loneliness

Baseline moderate/severe pain was associated with loneliness 4 years later, independently of age, sex, baseline loneliness, ethnicity, education, wealth, marital status, physical activity, and depressive symptoms (Table 2). The adjusted OR for overall pain was reduced from 1.86 in model 1 to 1.34 in model 4, indicating that baseline loneliness, sociodemographic factors, and depressive symptoms explained a substantial proportion of the association between baseline pain and future loneliness. Nonetheless, the OR of 1.34 indicates that among older people, pain does presage greater loneliness in the future. The full regression results from model 4 are detailed in Supplementary Table 3 (available at http://links.lww.com/PAIN/B182) and indicate that baseline loneliness, older age, female sex, lower wealth, unmarried status, and depressive symptoms were independently associated with future loneliness. The sensitivity analysis indicated that association between loneliness and follow-up pain remained significant after baseline mobility impairment had been included as a covariate (adjusted OR = 1.25, 95% CI 1.05-1.49, *P* = 0.012).

The analysis of incident loneliness on follow-up among people who were not lonely at baseline is detailed in Supplementary Table 2 (available at http://links.lww.com/PAIN/B182). In the fully adjusted model, the odds of incident loneliness were 1.58 (1.28-1.95), *P* <0.001, slightly greater than in the analysis of the full sample. Numerically, 15.9% of participants with no pain at baseline became lonely on follow-up, compared with 23.1% of those who were in pain at baseline.

### 3.3. Interactions with inflammation

**Figure 1.**
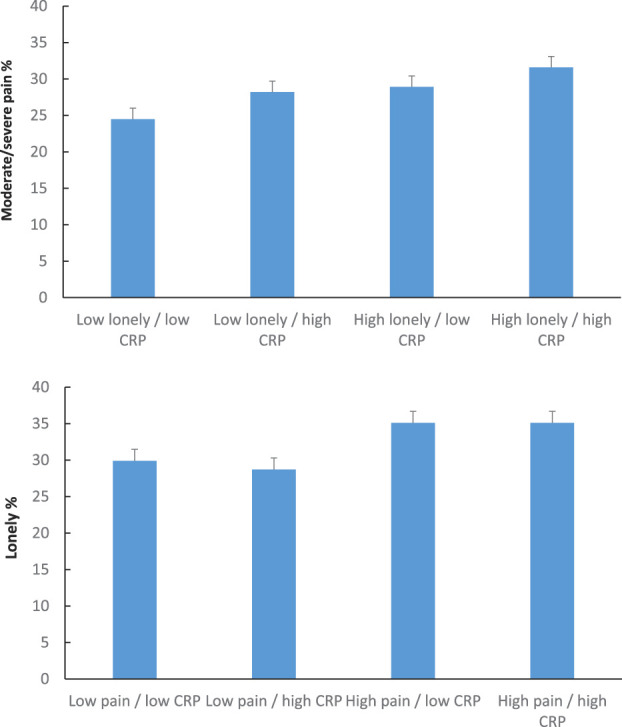
Upper panel: Proportion of individuals experiencing moderate/severe pain at 4-year follow-up in low lonely/low C-reactive protein (CRP), low lonely/high CRP, high lonely/low CRP, and high lonely/high CRP groups, adjusted for age, sex, baseline pain, ethnicity, education, wealth, marital status, physical activity, and depressive symptoms. Error bars are SEM. Lower panel: Proportion of individuals experiencing moderate/severe pain at 4-year follow-up in low pain/low CRP, low pain/high CRP, high pain/low CRP, and high pain/high CRP groups, adjusted for age, sex, baseline loneliness, ethnicity, education, wealth, marital status, physical activity, and depressive symptoms. Error bars are SEM.

We tested the involvement of inflammation in the bidirectional links between loneliness and chronic pain in the 3701 participants for whom high-sensitivity CRP was available at baseline. Baseline CRP was associated with both baseline pain (*r* = 0.13, *P* <0.001) and baseline loneliness (*r* = 0.034, *P* = 0.043). Longitudinally, CRP concentration predicted future pain (OR = 1.22, 95% CI 1.02-1.45, *P* = 0.029), but not future loneliness (OR = 0.97, 95% CI 0.81-1.16, *P* = 0.76). To test the contribution of inflammation in the relationship between loneliness and future chronic pain, we compared 4 groups defined by the presence of loneliness and elevated CRP at baseline. As shown in Table 3, 49.2% of participants were in the low lonely/low CRP group, and the smallest proportion was in the high lonely/high CRP group (9.8%). After all covariates had been taken into account, there was a significant interaction between loneliness-inflammation group and future chronic pain. Compared with the low lonely/low CRP group, chronic pain on follow-up was significantly more common in all other groups. Notably, there was a trend across groups (*P* = 0.003), with the highest odds of chronic pain in the high lonely/high CRP group (OR = 1.50, 95% CI 1.13-2.00, *P* = 0.006). These results are illustrated in Figure 1 (upper panel), where it is evident that 7.1% more individuals in the high lonely/high CRP group experienced moderate or several chronic pain on follow-up than did the low lonely/low CRP group, and the other 2 groups showed intermediate levels.

**Table 3:** Loneliness, chronic pain, and C-reactive protein.

	Grouping	%	Adjusted OR (95% CI)	*P*
Loneliness and CRP as predictors of future chronic pain	Low lonely/low CRP	49.2%	1 (ref)*	
	Low lonely/high CRP	21.9%	1.27 (1.03-1.58)	0.028
	High lonely/low CRP	19.0%	1.36 (1.07-1.71)	0.011
	High lonely/high CRP	9.8%	1.50 (1.13-2.00)	0.006
Chronic pain and CRP as predictors of future loneliness	No pain/low CRP	55.6%	1 (ref)†	
	No pain/high CRP	21.3%	0.93 (0.75-1.15)	0.43
	Pain/low CRP	12.7%	1.34 (1.04-1.73)	0.023
	Pain/high CRP	10.5%	1.35 (1.02-1.78)	0.036

CRP, C-reactive protein.

* Adjusted for age, sex, baseline chronic pain, ethnicity, education, wealth, marital status, physical activity, and depressive symptoms.

† Adjusted for age, sex, baseline loneliness, ethnicity, education, wealth, marital status, physical activity, and depressive symptoms.

The corresponding analysis that tested the combination of baseline pain and CRP in relation to future loneliness is also summarised in Table 3. In this case, pain was the primary determinant of future loneliness, and CRP concentration had no additional role. Thus, the adjusted ORs for baseline pain/low CRP and baseline pain/high CRP were almost identical (1.34 and 1.35 respectively). As can be seen in Figure 1 (lower panel), future loneliness was more common among participants who reported moderate/severe pain at baseline compared to those with no pain. It seems therefore that inflammation augmented the association of baseline loneliness with future pain, but did not play a role in the link between baseline pain and future loneliness.

## 4. Discussion

These analyses examined the bidirectional associations between pain and loneliness over a 4-year period in a large population sample of older men and women. We found that loneliness at baseline predicted moderate/intense pain on follow-up, and that pain at baseline was related to later loneliness, confirming that there are 2-way associations. The links were explained in part by demographics, physical activity, depressive symptoms, and mobility impairment, but even when these factors had been taken into account, the associations remained robust. Analyses of a subsample in which CRP was measured suggested a different role of inflammation in the 2 directions of association. Higher levels of inflammation seemed to augment the relationship between baseline loneliness and future pain, such that the combination of loneliness and inflammation conferred a greater risk of pain on follow-up than either factor individually. By contrast, inflammation did not play a role in the association between pain at baseline and future loneliness.

The prevalence of moderate/severe pain increased slightly between baseline when participants were aged 65.1 years on average, and 4 years later, with a larger increase among women (3.9%) than men (2.7%). Loneliness increased by only a modest extent on average, as has been observed previously.^41^ However, there were shifts in both directions in a substantial minority of respondents, with increases and decreases in pain and loneliness over time. The strongest predictors of future pain and loneliness were baseline levels of the 2 experiences. There was a 6-fold increase in risk of pain on follow-up for people who were in pain at baseline, whereas lonely individuals at baseline were 7 times more likely to be lonely 4 years later. Notably, the strength of associations between baseline loneliness and future pain, and baseline pain and future loneliness, was very similar, with adjusted ORs of 1.25 and 1.34, respectively. The other factors entered into the statistical models also explained similar proportions of the 2 relationships. Computing the proportion of association explained each set of factors,^24^ it is apparent that 22% of the association between baseline loneliness and future pain was explained by sociodemographic factors (ethnicity, education, and wealth), marital status, and physical activity. Analogously, 24% of the link between baseline pain and later loneliness was explained by these same factors. The pattern reinforces the close interdependence of pain and loneliness in this sample. One influential theory argues that societal exclusion activates brain regions also responsible for processing physical pain.^10^ It is suggested that evolutionarily, pain computations responsible for preventing danger were adopted by social attachment systems to trigger in response to social separation to avoid harmful consequences,^25^ creating a centrally driven association between loneliness and physical pain. However, the direct overlap between neural processes implicated in physical and social pain is uncertain, and the association may reflect more fundamental motivational pathways.^15^

Although the associations between loneliness and later pain parallel previous research,^19^ the longitudinal results for pain being related to later loneliness were more robust than those of other population studies. For example, analyses of the Health and Retirement study reported by Emerson et al.^12^ did not find that baseline pain predicted future loneliness, although the combination of pain both at baseline and follow-up was related to loneliness. Another study of a community sample from Arizona found minimal associations between pain intensity or pain frequency and loneliness measured 6 to 53 months later.^4^ Differences may relate to the definitions of pain and measures of loneliness used in these investigations, and in the selection of covariates.

There was some overlap between the other variables included in these analyses that were related both with future pain and future loneliness. Female sex, lower socioeconomic status defined by wealth, and depressive symptoms were associated with both directions of the pain–loneliness relationship. This reflects the greater vulnerability of these groups to chronic pain and loneliness. By contrast, physical inactivity was a risk factor for future pain but not loneliness, whereas older age and being unmarried were associated with subsequent loneliness but not pain. The role of physical activity in protecting against episodes of future pain has been established.^35^ Loneliness is also correlated with physical inactivity cross-sectionally, but there is less evidence for longitudinal associations at older ages.^21^ Increasing age and not having a marital partner are recognised risk factors for loneliness, whereas the links between marital status and pain are more complicated, and vary with the nature and quality of marital interactions.^22^

Our analyses involving inflammation identified some differences between the 2 directions of association. These analyses were conducted on a smaller sample (3701 compared with 4906) because CRP was obtained on a separate occasion from the main assessments. Not all participants had a study nurse visit, and blood samples were not obtained from everyone because of the presence of clinical factors such as clotting disorders, or refusal. However, these analyses suggest that inflammation may play a role in the relationship between loneliness and future pain, but not in the association between baseline pain and future loneliness. This is apparent in Table 3, where the adjusted OR for people in the high lonely/high CRP group (1.50) was greater than for the high lonely/low CRP or low lonely/high CRP groups (1.36 and 1.27, respectively), with a significant trend across groups. Loneliness is associated with proinflammatory gene expression, with heightened inflammatory responses to stress, and with raised IL-6 concentration.^8,17,33^ Not all lonely people express heightened inflammation, but the combination seems relevant to future pain experience. This is not to imply that inflammation is the only or even the key pathway linking loneliness with pain. It has been argued that social isolation increases pain inference and pain intensity mediated by pain vigilance, the abnormal focus on signals of pain and potential injury.^20^ Furthermore, loneliness may increases pain sensitivity, thereby amplifying the individual's future pain experience.

By contrast, CRP was not relevant to the association between baseline pain and future loneliness. The increased likelihood of being lonely on follow-up was the same when baseline pain was coupled with lower or higher CRP concentrations. Although there was a cross-sectional relationship between CRP and loneliness at baseline, CRP concentration on its own was not associated with future loneliness. This indicates that other biological, social, or psychological pathways not involving inflammation are relevant to the link between pain and later loneliness. Several factors may be involved. For instance, the experience of moderate/severe pain may curtail social activity, involvement in family events, and reduce cultural engagement, all of which are protective against future pain.^13,27^ People with chronic pain can feel misunderstood when loved ones judge or ignore the suffering person's feelings, promoting a sense of isolation.^40^

This study has a number of limitations. Although loneliness was assessed using a standardised questionnaire, pain was measured with a single item concerning pain intensity, and duration of pain experience was indexed by asking participants whether they were “often” troubled by pain. The study is observational, and we took into account a wide range of sociodemographic, behavioural, and emotional factors that potentially confound the links between pain and loneliness. However, there may be unmeasured factors that explain the association, so causal conclusions cannot be drawn. Only a small proportion of the sample were of non-white European origin, so results may not generalise to other sectors of the population. It should also be noted that neither pain nor loneliness is the primary focus of assessments in ELSA, reducing the possibility of expectations concerning their relationship influencing responses.

In conclusion, this analysis of a large representative population sample confirmed bidirectional associations between loneliness and pain over a 4-year period. The magnitude of associations was similar in the 2 directions, and remained after adjusting statistically for demographic factors, physical activity levels, and depressive symptoms. Nonetheless, we found some distinctions between the mechanisms involved in these relationships, with systemic inflammation playing a more prominent role in the association between loneliness and subsequent pain than in the link between pain and later loneliness. This suggests that the pathways responsible for the associations differ. Both pain and loneliness are distressing experiences that impact well-being and quality of life. Greater understanding of these bidirectional relationships may help identify methods for breaking these links.

.

## Conflict of interest statement

The authors have no conflicts of interest to declare.

## Appendix A. Supplemental digital content

Supplemental digital content associated with this article can be found online at http://links.lww.com/PAIN/B182.
